# Supplementary material for: Are carbon emissions trading and green financial instruments synergistic? -Comprehensive quantitative research based on content analysis
Source: PLoS One. 2024 Mar 7;19(3):e0298601. doi: 10.1371/journal.pone.0298601 (PMC10919601; doi:10.1371/journal.pone.0298601)
Supplement: S1 Data — (ZIP) [file pone.0298601.s001.zip › Data/地级市Z_F工作报告.zip!/║┌┴·╜¡/┤≤╟∞╩╨/└┤╫╘╣1⁄2╓┌║┼í╛┬φ┐╦╩2╛▌═°í┐_2013.docx]

各位代表：

　　现在，我代表市人民政府向大会报告工作，请予审议，并请市政协委员和列席会议的同志提出意见。

　　一、2012年工作

　　过去一年，我们围绕建设现代化国际化城市战略目标，按照市第八次党代表大会、市委八届二次全会要求部署，以新的更高标准狠抓落实，全面完成了市九届人大一次会议确定的各项任务。

　　（一）经济持续健康发展。在国内经济下行压力加大的形势下，预计全市地区生产总值突破4000亿元、增长10%以上、

　　实现与全省同步，油与非油经济比重46:54；地方财政收入263亿元、增长24.8%；固定资产投资1375亿元、增长31%，社会消费品零售总额800亿元、增长20%，万元GDP综合能耗下降3.9%。工业规模效益显著提升。完成增加值3150亿元、增长10%，其中地方工业增长20%，战略新兴产业增速和规模全省第一。原油连续10年保持高水平的4000万吨稳产，再次创造了同类油田开发建设史上的奇迹；120万吨乙烯改扩建、60万吨聚丙烯二期等建成投产，产能均进入全国同行业前列；忠旺新型铝材等龙头项目加快建设，沃尔沃整车基地项目开始设备安装调试，伊利六期4.5万吨配方奶粉等项目达产达效；高新区综合排名上升到全国第29位，经开区晋升为国家级经开区，德力戈尔和杏山园区升级为省级园区，全市园区工业占地方工业比重提高到70%。现代农业迈上新台阶。完成增加值153亿元、增长15%。粮食产量130亿斤、增长17%，实现“五连增”，综合生产能力居全省前列；“三退三进”全面推开，工厂化、标准化、规模化养殖比重提高到60%，带动畜牧业发展方式加快转变；棚室新增6万栋、总量20万栋、果蔬产量30亿斤，冬季果蔬自给率达到60%。服务业呈现快速发展的好态势。完成增加值610亿元、增长15%。继万达广场之后，华联商厦、居然之家等品牌店投入运营，新兴便民商业网点增加3000个；旅游知名度不断提高，本地人游起来、外地人多起来，旅游收入增长30%；新华（大庆）国际石油资讯中心启动运营，新肇内陆港实现铁海联运，招商银行等7家金融机构入驻，银企对接120亿元贷款合同全部落实，企业债券成功发行，服务业助推制造业、繁荣城市和辐射区域功能明显增强。（二）城乡功能品质加快提升。顶层规划设计更加完善。修编《大庆市城市总体规划》，编制完成《大庆现代化国际化城市空间发展规划》、《总体城市设计规划控制导则》，进一步确定了城市总体定位、布局和品质等顶层战略，突出“五线”控制，解决建筑高度、密度、风格等十个关键问题，规划前瞻性、科学性和权威性明显增强。城市基础功能进一步提升。实施重点建设项目203个，完成投资384亿元，新增建成区7.8平方公里。铁路东西两站、国际会议中心、奥林匹克公园等重大工程顺利推进，油立方迁建等36个城建大项目竣工，区域中心城市功能加快完善；城区70条道路竣工通车，打通了中八路、经一街等瓶颈路和断头路，建成了北一路、南一路两条全程立交的快速干道，东西城区之间行车时间缩短一半以上，“两环四纵四横”现代城区路网体系基本形成；农村新造林15.8万亩，城区新植树1000万株，建成区绿化覆盖率达到45.2%。让胡路区和杜尔伯特成为全省首个省级生态区和生态县。小城镇和示范新村建设步伐明显加快。连环湖旅游名镇、朝阳沟镇等经济重镇的承载功能进一步增强，湖韵新村等16个示范新村建设稳步推进，产业要素和周边人口加快向重点村镇集聚，对促进城镇化、拉动区域经济和创新农业经营体制的带动作用日益显现。城市管理水平不断提高。深入开展城乡环境综合整治，新开发区块和新建道路等管理体制进一步理顺，3500个网格单元全部实现定位定人定责，市区重点要害部位实现视频监控全覆盖，主城区主次干道保洁清雪机械化作业率提高到70%。继国家环保模范城通过新国标复检之后，国家卫生城又顺利通过复审，全国文明城市指数测评列127个参评城市第17位。

　　（三）改善民生力度明显加大。城镇居民人均可支配收入和农村居民人均纯收入分别达到25425元和11500元、增长13%和24%，均高于GDP增速。年初确定的10个方面民生实事全部落实。社会综合保障更多地惠及百姓。城镇新增就业4.4万人，大庆被评为全国创业先进城市；“五险”扩面净增8.4万人，新农保和城居保人数达到49万人，新农合人均筹资额提高25%，企业离退休人员工资人均增长14%，市区最低工资、城市低保标准分别提高32%和12%；改造农村泥草房1.6万户，建设保障性住房180万平方米，城乡10万多人口改善了住房条件。城乡公共服务更好地满足需求。教育事业更加均衡，新建改建城乡公办幼儿园90所、农村寄宿制学校12所，总数分别达到255所和55所，大庆成为全省惟一的全国“两基”工作先进地区；医疗卫生更加优质，新建改建村卫生室183个，8所社区卫生服务中心被授予国家或省级示范中心，油田总医院、市人民医院成为省级区域性诊疗中心，市级医院与国内外70所名院714位专家协作共建、三级医院领办社区卫生服务、县乡村医疗机构一体化管理的新格局基本形成；文化事业更加繁荣，新建改建112个社区和村综合文化场所，舞剧《鹤鸣湖》等72部文化作品荣获“五个一工程”奖等省级以上奖项，话剧《大湿地》作为“十八大”献礼剧目进京演出，市博物馆陈列收藏的东北第四纪古生物化石种类和数量居全国之首。以“五有”为标志，大庆精神、铁人精神教育正式纳入国民基础教育体系，对于青少年价值观形成和城市人文精神传承产生了重要影响；养老服务更加完善，新增老年人日间照料室40个、总数达到226个，新增养老机构9家、总数达到93家，社区为老服务功能日趋健全，基本形成公办引领、民办补充、居家为主的社会化养老服务体系；城市公交更加便捷，新开通东西城区快线等公交线路8条、增投公交车辆550台，针对冬季雪大天冷实际，加密班次、延长营运，缩短了群众等车时间。（四）改革开放扎实推进。重点领域改革有新突破，探索创新基层社会管理体制，启动推行“一委一站一会”模式，将市区29个街道和200个社区整合为70个新社区，取消街道层级，扁平化管理，由“线”向“块”集中，为加强社会建设打下坚实基础；深入推进医药卫生体制改革，全面推行基本药物制度，出厂价格销售的380种药物覆盖社区和乡村卫生院所，减轻了群众医疗负担；进一步深化文化体制改革，歌舞团等6家文化院团转企改制，文体旅集团顺利组建，图书、出版、音像等单位企业化运营，大庆被评为全国文化体制改革先进地区；落实农村土地管理制度改革，全面完成128万公顷农村集体土地确权，为推进农村集体土地流转、土地征收和土地整治奠定了基础。行政审批制度、财政投融资体制等项改革也取得新的进展。对外开放有新进展，全市招商引资到位资金262亿元、增长21%；外贸进出口总值28亿美元、增长30%，实际利用外资5.03亿美元、增长19%；拓展对外交流，成功举办2012北京·大庆文化艺术周，城市形象广告片继投放央视之后又亮相纽约时报广场“中国屏”，大庆入选“2012世界特色魅力城市200强”。

　　（五）社会管理切实加强。社会协调机制运行顺畅有力，成立群众工作中心，建立民情社情政情发布制度，建设互联网舆情研判导控中心，健全“六调联动”体系、“四级”群众工作网络和领导干部接访包案等制度，全年调解各类矛盾纠纷3万件次，调处成功率达98%。特殊人群服务管理取得重要进展，在全省率先推行居住证制度，流动人口在住房保障、教育收费、医疗服务等方面享受市民待遇，20万新市民有了家的归属感；全年免费收治家庭无力管护、有肇事肇祸倾向的精神病人500人次，建设过渡性安置中心7个，接收管理和安置刑释解教人员、社区矫正对象6600人次。社会保持和谐安定，持续加强“八大领域”安全监管，深入开展“打非治违”和“打四黑除四害”专项行动、“打黑除恶”专项斗争，八类主要刑事案件下降10.3%，生产事故下降49.8%，市民安全感提高到98.4%。大庆成为首批25个全国质量示范城市之一。

　　各位代表，过去一年，现代化国际化城市建设实现良好开局，得益于市委的坚强领导，得益于人大、政协的有力监督，得益于各民主党派、工商联、无党派人士和群团组织的积极参与，得益于石油石化大企业及驻庆中省直单位的鼎力支持，得益于老领导、老同志的关心帮助，得益于全市广大干部群众的团结奋斗。在此，我代表市政府，向全市人民，向所有为大庆经济社会发展做出贡献的同志们、朋友们，表示崇高的敬意和衷心的感谢！

　　回顾过去一年，我们在看到成绩的同时，也清醒地认识到差距和问题。主要是：对照建设现代化国际化城市内在要求，地方经济产业质量、结构、布局和层次有待优化，尤其是服务业比重不高，需要加快由满足域内向服务区域转变；市政基础设施承载能力与城市快速发展不相适应，区域中心城市功能相对不强，需要加快由基础功能补位向整体品质提升转变；城市规划建设管理有薄弱环节，存在监管不到位的问题，需要加快由集中整治向常态规范管理转变；城乡发展存在差距，公共服务尚不均衡，需要加快由局部优质向全域等质转变；个别部门和公务人员服务意识不强、工作作风不实，政府职能需要进一步转变。二、2013年工作安排

　　2013年是深入贯彻落实党的十八大精神的开局之年，也是提前五年建成全面小康社会、完成建设现代化国际化城市第一步目标的关键之年。市第八次党代表大会提出了“大庆建设现代化国际化城市”的宏伟目标，市委八届二次全会明确了“加快建设全面现代化专业国际化城市”的战略定位，标志着大庆发展迈上新起点、踏上新征程。党的十八大确定全国到2020年建成全面小康社会，我省确定提前三年建成全面小康社会，我市确定提前五年建成全面小康社会，目前实现程度为85.2%，人均GDP、城乡居民收入比等18项主要指标已达到或接近标准，第三产业增加值、科研经费支出、文化产业等5项指标还存在差距，大庆建成全面小康社会进入攻坚期。为此，我们必须坚定不移地实施“一二三四五”发展战略，一以贯之地坚持全党抓发展，中心抓经济，重点抓项目，努力打牢产业、市政、社会、人文和体制五个基础；我们必须加快调整经济结构和转变发展方式，不断提高公共财政收入占GDP、税收占财政收入、产业投资占固定资产投资、非公经济产业投资占产业投资、民生投入占财政支出五个比重，努力实现速度、质量和效益协调统一；我们必须致力于求实务实落实，把困难考虑得更充分一些，把办法想得更周全一些，把工作做得更扎实一些，牢牢把握发展主动权，加快把美好愿景变成现实。

　　今年政府工作总的要求是：深入贯彻落实党的十八大精神，按照市委八届三次全会部署，紧紧围绕建设现代化国际化城市和提前五年建成全面小康社会目标，以科学发展为主题，以加快转变经济发展方式为主线，着力提高经济增长质量效益，着力统筹城乡建设，着力保障改善民生，着力促进社会和谐，着力强化体制创新，进一步打牢“五个基础”，努力实现更好更快更大发展。主要预期目标是：地区生产总值增长11%，固定资产投资增长30%，社会消费品零售总额增长15%，地方财政收入增长20%，城镇居民人均可支配收入和农村居民人均纯收入分别增长13%和15%，万元GDP综合能耗下降3.5%。

　　（一）提高经济增长质量效益，构建现代产业体系，进一步打牢产业基础。深入实施“5·10·15”计划，坚持抓项目抓企业抓品牌，不断优化产业布局，打造高新区高新技术产业，萨尔图区、让胡路区和龙凤区主城区现代服务业，红岗区和大同区城郊经济，杜尔伯特和林甸县生态经济，肇州县和肇源县现代农业等地方经济“五大特色板块”，加快构建以高科技含量、高附加值、低污染、低能耗、自主创新为特征的现代产业体系。全年开工建设千万元以上产业项目330个、其中亿元以上160个。稳油增气，壮大新兴产业，做优做强工业。工业增加值增长10%，其中地方工业增长20%。全力服务油田产能建设。维护油田生产秩序，支持油田精细开发，稳定原油产量，增加天然气产能，确保原油4000万吨稳产，加快向油气当量5000万吨目标迈进。合力推进石化产业延伸增值。围绕重油轻质化利用和石化深加工两大方向，延伸丙烯、乙烯、芳烃等六大产业链条，与石化大企业和战略投资者合作，携手争取原料加工指标，谋划建设15万吨丙烯、120万吨燃料油综合利用等10个大项目，确保50万吨c综合利用等项目投产达效。大力推动新兴产业加速发展。确保沃尔沃汽车年内实现批量生产，鑫龙江新能源客车项目具备整车生产条件，同步配套建设汽车零部件产业园，力促汽车产业整体突破；确保忠旺硬合金生产线达到设备安装条件，配套规划建设金属材料深加工产业园，佳昌砷化镓抛光片产能由40万片扩大到80万片，力促新材料产业成长壮大；确保航天三沃高效能油田输送装备等项目投产，积极引进中国机械工业集团等战略投资企业，力促装备制造业提档升级；确保低碳光伏产业园高晶硅电池片和太阳能光伏组件一期投产，加快建设大唐等风电项目，力促新能源产业规模扩张；确保单克隆抗体药物等研发成果实现产业化，完善福瑞邦生物科技园综合功能，力促生物产业集聚发展。

　　突出粮食、果蔬、畜牧“三位一体”格局，加快发展现代农业。农业增加值增长15%。扶持种养大户、家庭农场、专业合作社等新型经营主体，着力提升集约化、专业化、组织化和社会化水平。加快国家现代农业示范区建设，重点建好200万亩沿江水稻产业带和600万亩高产玉米产业带，新建农机专业合作社100个，力争粮食产量达到145亿斤；统筹推进棚室经济，重点建好林甸四季青温泉果蔬、红岗北极花谷、大同航天育种果菜等20个基地，建设大同农产品物流园、林甸果蔬物流中心和肇州果蔬仓储交易中心，促进棚室建设、果蔬生产和市场销售同步推进、链式发展；加快实施畜牧业养殖“三退三进”，抓好伊利万头奶牛国际化标准牧场、正邦百万头生猪基地和30万只蛋鸡养殖基地等项目建设，新建改建养殖牧场150个，标准化规模养殖比重提高到70%。同时，依托丰富河湖资源，大力发展绿色生态水产养殖加工产业。

　　立足市域，辐射区域，加速壮大现代服务业。服务业增加值增长15%。坚持“随着城市进程走、迎着生活需求上、围着生产服务抓、跟着深度创意谋”，推动生产性服务业由自我服务向区域辐射、生活性服务业由保障基本需要向满足多元需求方向转变。推动金融产业集聚发展，支持现有金融机构创新服务产品，大力引进银行、证券、保险、风投、创投等金融机构，争取发行第二期企业债券，提升金融对经济发展的支撑力；推动信息服务产业突破发展，壮大服务外包业，争取国家石油能源价格信息发布中心移至大庆，建设联想科技城云计算、物联网和移动互联中心，引建爱立信、百度等关联企业，扩大新一代信息技术应用领域，加快建设智慧城市；推动物流产业扩张发展，重点建好新肇内陆港、太阳升综合物流园，畅通铁海联运和江海联运通道，推进昌升国际商贸城等龙头项目，打造区域性生产物资、工业原料和建筑材料集散中心；推动商贸产业升级发展，扶持规范电子商务等新兴便民业态，推进“社区商业双进”工程，加快建设唐人中心、王府井百货、松雷广场等重点项目，助推大商、万达提升域外辐射能力，带动形成东部城区、西部城区和萨尔图区“三大核心商圈”；推动文化产业繁荣发展，大力发展影视传媒、新闻出版、演出娱乐、休闲健身等重点产业，建设百湖艺术群落二期、“9·26”艺术区等重点项目，搞好油立方、奥林匹克公园、黑鱼湖国际艺术村等功能载体运营；推动旅游产业跨越发展，围绕建设旅游目的地城市，打造全域全业全季全民旅游，释放石油文化、湿地风光、草原风情、温泉养生等资源组合优势，创意开发特色旅游产品，完善连环湖、鹤鸣湖等主要景区综合配套功能，推进北国之春梦幻城、阿木塔蒙古风情岛等重点项目，扩大湿地旅游文化节、雪地温泉节对外影响力，旅游收入增长30%。（二）统筹城乡发展，建好宜居宜业家园，进一步打牢市政基础。强化顶层设计引领建设功能设施，推进“四城”联创牵动提升现代管理水平，突出“集约智能绿色低碳”方向推动城乡品质升级。

　　严格执行规划设计，全域对接现代化国际化城市建设标准。依据《大庆现代化国际化城市空间发展规划》等顶层设计，编制完善控详规划，严格审批修详规划，做好产业布局、土地利用、道路交通、市政设施等专项规划，把握好城市发展方向。着眼建设“城市拓展区”，重点推进新城开发。庆东新城突出阿斯兰小镇、龙凤小镇、湿地公园等精品工程建设，打造城市靓丽门户；庆北新城突出完善兰德湖地区建设，综合治理王家围子区块环境，全面提升承载功能；庆南新城突出改造治理旧城区，高标准拓展组团开发规模；庆西新城完成富强地区改造任务，推进西客站周边地区整体开发。着眼建设“市郊功能提升带”，重点做强新型城镇。统筹产业支撑、功能服务、人口转移和生态环境，加快龙凤镇、喇嘛甸镇、杏树岗镇城市化步伐，扩大连环湖、四季青等旅游名镇影响力，扩展肇源镇、新站镇、太阳升镇的物流通道功能，加快构建以城关镇为牵动、特色经济重镇协同的县域经济多元支撑和城镇化多点带动新格局。着眼建设“外围城镇发展廊道”，重点建好示范新村。完成肇州实现村、大同民强村等16个示范新村建设任务，同步推进农村环境综合整治，统筹提升城镇化质量和水平。依法严格执行规划，加强“五线”管理，解决好“十个关键问题”，继续开展城乡规划建设管理领域问题综合治理，确保建设项目符合规划布局、符合设计标准、符合质量要求。

　　坚持建管并重，整体推动城乡环境功能加快提升。让城乡道路更加畅达，升级乡村公路82公里，改造城区道路13条，健全城乡道路交通标识等配套设施，特别是打通南三路至南五路的东干线段、南三路至凤十四路段、南一路至凤阳桥段、北一路与新村北环连接处等4个节点瓶颈，最大限度释放“两环四纵四横”快速路网的通行效率；让基础设施更加完备，建好北城热源、东城区污水处理厂改造等“三供两治”项目，增加公园、公厕、公交站亭等公共便民设施，建设市职工服务中心、市福利中心、驾驶员考试中心等功能项目，充分保障和方便市民生活；让城市管理更加高效，深入实施“三四五六”工程，持续开展城乡环境十项综合整治，突出抓好机场路等沿线沿街、薄弱区块和重点部位细部整理，明确规范网格单元的管理责任、内容和标准，城区新建视频监控点3000个、总数达到1.4万个，充分发挥监控系统综合管理功能，加快提升现代城市管理水平。

　　强化产城融合，统筹促进建城与兴业协调发展。完善重大基础设施功能，扩建萨尔图机场打造国际空港，建成铁路东西两站形成重要交通枢纽，配合省里推进哈大齐客运专线、大绥高速、明沈公路肇州绕行线改建，加快华能热电联供、东城污水处理厂再生水深度处理等重点项目建设，增强产业发展保障能力；升级园区产业承载功能，重点抓好高新区、经开区、林源工业区的铁路专用线建设，完成后四厂和让库搬迁，搞好301运河沿线地区综合开发，专业化市场化运作建设园区公共技术、企业孵化、检验检测等服务平台；配套关联服务功能，建设城市综合体，打造特色餐饮娱乐街区，加快星级宾馆及影院、酒吧、健身休闲等场所建设，优化公交站点和线路，推动吃住行游购娱功能整体提升。

　　建设美丽家园，系统优化生态环境。持续推进植树、复草、治水、净气、降噪，重点对城乡干路、居住小区、油田厂矿、乡镇村屯进行全方位绿化和细部绿地升级，农村造林15.3万亩，改良治理“三化”草原20万亩，启动建设森林植物园，城区植树1000万株、新增绿化面积1500公顷，营造“半城绿色半城楼”的优美意境；管好水源提升水质，优化水环境，加快东城水厂二期、应急备用水源地项目建设，综合治理萨北湖等6个湖泊，推进龙凤湿地水体污染防治，加强市域范围湿地综合保护；强化工业、农业、交通和城镇生活等污染源减排，市区实时监测PM2.5，力保新增3个省级生态县区，加快建设国家生态园林城市。（三）保障和改善民生，创新社会管理，进一步打牢社会基础。全面落实市委确定的基础设施、城市交通、社会服务、卫生教育、文化建设、生态建设等六大类“百项民生项目”，确保在学有优教、劳有丰酬、病有良医、老有善养、住有佳所等方面取得新进展，全力构建和谐大庆。

　　改善群众基本生活。扩大就业促进增收，做好以高校毕业生为重点的青年就业和农村转移劳动力、城镇困难人员、退役军人就业工作，全年新就业5万人。健全收入持续增长机制，确保城乡居民收入增长与经济发展同步；保持物价总体平稳，综合施策平抑物价、调控房价，规范物业等服务价格，扩大“平价商店”覆盖，尽可能减轻居民基本生活压力；提升综合保障水平，促进社会保障从制度全覆盖向人群全覆盖转移，新农保和城居保参保人数扩大到51万人，适时提高城乡居民最低生活保障标准，推动城乡医疗保险市级统筹，建设保障性住房150万平方米，解决好老居住区立面破损、管网老化、房屋渗漏等问题。

　　提升城乡社会服务。继续整合优化教育资源，逐步推行中心村以上办小学、乡镇以上办初中、县以上办高中，县乡村统筹布局幼儿园。按规划撤并农村学校15所，标准化新建改建农村寄宿制学校10所、城乡公办幼儿园90所，建设大庆市外国语学校、实验中学二部，建成二十三中和四中新校区，异地改建交通技校，扩建八一农垦大学，迁建东北石油大学艺术学院，统筹发展好各级各类教育；整体提升医疗水平，新建扩建170所乡镇卫生院和村卫生室，实现标准化基层卫生机构全覆盖，建成市妇女儿童医院，推广社区“健康小屋”，探索建立家庭医生制度，使城乡居民享受方便优质的诊疗服务；抓好养老服务标准化建设，新建养老机构10家以上，建立养老机构准入和服务质量标准，制定居家养老服务规范，综合提升社会化养老服务水平；进一步改善停车和出行条件，通过施划泊位、建设平面停车场和立体车库等措施新增停车泊位10万个，新开通公交线路4条、总数达到67条，更新投放公交车辆300台、总数达到2300台，主城区公交实现全覆盖。强化交通科学组织，规范出租车行业秩序，搞好交通环境综合治理。

　　强化社会管理。有效防范和化解社会矛盾，健全重大决策社会稳定风险评估机制，建好群众工作中心，完善“六调联动”工作体系，依法公平解决征地拆迁、劳资矛盾等“十个方面重点问题”，加强刑释解教、有不良行为青少年等特殊人群常态化服务管理，最大限度减少不和谐因素；保障城市公共安全，提高应急救援反应能力，保障水电气暖稳定运行，强化食品药品质量现场检测，完善城乡中小学校安全设施，保持“打四黑除四害”和“打非治违”高压态势，抓好危险化学品、道路交通、工程建设、消防安全等“八大领域”安全监管，努力创建国家安全发展示范城市。

　　抓好社会动员。大力发展社会组织，建立市级社会组织孵化基地，培育大庆爱心企业家联盟等10家以上全市性社会组织，加强行业协学会建设，更好地承接政府转移和委托的社会服务项目；扩大社会公众参与，落实民情社情政情发布制度，建立市民网络问政平台，发挥好市民议事厅、行风热线等载体作用，畅达民意，畅通舆情；强化社会心态引导，更加注重人文关怀，引导群众形成理性平和的心理预期，增强通过勤劳致富改善生活的主流理念，形成改善民生既需要党和政府努力，也需要人民群众自身奋斗的共识，为建设现代化国际化城市提供正能量。　（四）彰显文化自信，增强城市软实力，进一步打牢人文基础。深度挖掘特色文化资源，兼容并蓄现代文化元素，促进人文精神导引、公共文化服务、文化产品开发的融合互动，建设文化强市。强在城市之魂。弘扬大庆精神、铁人精神，建设核心价值体系，形成信守使命、担当责任、艰苦奋斗、务实创新的人文品质，筑牢大庆人文根脉；在国民基础教育体系中落实“五有”保障，强化大庆精神、铁人精神对青少年的价值观影响，使这一宝贵精神财富永续传承；建设大庆党史馆、廉洁文化主题公园，与铁人王进喜纪念馆、油田历史陈列馆、城市规划展示馆、博物馆等共同形成具有鲜明大庆特色的场馆教育基地，涵养文化底蕴，增进大庆人的文化自觉。强在市民素质。倡导“十大观念”，整改“十大陋习”，养成文明生活方式，特别是注重从遵章驾驶等行为细节上引导市民强化讲规矩、重规章、守法纪的规则意识，从节约一滴水等日常习惯上引导市民形成珍惜资源、爱护环境的生活态度，从帮助身边人做一件好事等具体行动上倡导形成互信互助、和谐友善的社会风尚，让文明植根心底、走进生活。强在文化服务。新建改建50个社区和村文化活动室，确保市图书馆、西城区青少年科技文化活动中心等建成投用，推动现有文体场馆向社会有序开放，最大限度释放文化设施服务功能；挖掘利用冰雪、湿地、猛犸象化石等特色文化资源，创意开发《百湖传奇》等系列影视动漫作品，搞好黎明河休闲街区等策划运营，引导愉悦健康的文化消费；深入开展“激情之夏”、“书香大庆”等群众文化活动，鼓励发展票友会、车友会和乒乓球、自行车等俱乐部，办好大庆地企联合体育大会，带动全民健身活动广泛开展。

　　（五）推进改革扩大开放，持续增添发展活力，进一步打牢体制基础。扎实推进重点改革。深化政务公开，突出公共服务、行政审批等重点领域，收费、处罚、许可等重点环节，及时公开各项流程、依据、标准和时限，以公开促公平，以透明促监督。依法推进市级相关事权向县区、社区、园区下放，优化权力配置，提高行政效能；强化新社区管理服务，以开展“规范社区建设年”活动为牵动，完善落实社区相关管理和执法职能，地企联合建立60个社区服务综合体，打造“一刻钟社区服务圈”，努力在综合执法、多元服务上实现突破；提升城市管理市场化程度，扩大环境卫生保洁、园林绿化养护、市政设施维护等领域作业市场化，进一步提高管理效益和水平；探索创新产业园区管理机制，坚持“机构随着产业设、职责依据产业分、人员围绕产业抓”，高新区设立石化产业办、汽车产业办、新材料产业办等机构，其它园区根据产业定位参照建立相应机构，加大专业化集中扶持产业发展力度。同时，继续推进农村管理制度、医药卫生体制等改革取得新成效。进一步扩大对外开放。提升经贸合作水平，重点在与国内外知名商协会合作上突破，加大招商引资力度，引进亿元以上项目60个、到位资金180亿元。争取机场航空口岸纳入国家建设规划，扩大石化、机电等优势产品出口比重，外贸进出口总值增长20%。支持各类园区加强对外区域合作，加快发展“飞地经济”；提升区域影响力，策划举办国际石油石化技术与装备展览会、国际雪地狂欢节，升级办好汽车文化博览会，利用场馆资源积极申办各级各类体育赛事和文化展演；提升城市美誉度，继续强化海内外主流媒体宣传，加大城市形象广告投放力度，拓展与友好城市等城际间的交流合作，把城市的发展变化和人文魅力宣传出去，吸引更多的人流物流资金流汇聚大庆。各位代表，完成全年各项发展任务，必须坚持市委统一领导，主动接受人大法律监督和工作监督，自觉接受政协民主监督，广泛听取各界意见建议，切实加强政府自身建设。强化思想建设，始终坚持“负责任地干事、干负责任的事”工作理念，持续推进“政务文化建设工程”，引导激励政府公务人员，心里时刻装着建设现代化国际化城市的历史重任，忠诚信守责任使命，把实现人生价值融入到大庆建设发展的宏伟实践中；心里时刻装着百姓的安危冷暖，倍加关切群众忧为何事、难在何处，用实际行动践行为民宗旨；心里时刻装着发展中的问题和差距，勇于攻坚克难，善于突破创新，不断创造新业绩，实现新作为。强化制度建设，完善行政权力运行规范，提高依法管理城市的能力水平，做到职权法授、程序法定、行为法限、责任法究；完善工作推进保障机制，落实“五定五保”、“三位一体”督办、并联审批等有效举措，坚持现场办公和集中会办制度化常态化，及时研究解决现实矛盾和问题；探索建立标准化电子政务模式，加快政府办公自动化建设，持续推动行政提速、管理创新、执行彻底、结果问效。强化作风建设，严格落实中央改进工作作风、密切联系群众的“八项规定”，立足“两服务、两提高、两端正”，牢固树立终身学习理念，确保思维视野和能力本领适应发展需要；加强调查研究，实行周五调研日制度，时间可串不可占，深入机关企事业单位、项目现场、社区村屯，做到带着问题下、拿出办法回、解决效果好；精减政府系统会议、文件、简报，严控会议数量和规模，规范文件和简报制发。强化廉政建设，认真落实“一岗双责”，深入搞好廉政风险防控，严格自律，做好表率；改进完善项目招投标办法，强化工程发包、政府采购等重点领域监管，突出保民生、保发展，提高财政资金使用效益；发扬艰苦奋斗优良传统，厉行勤俭节约，反对奢侈浪费，务求干部清正、政府清廉、政治清明。

　　各位代表，新的一年，群众有很多新期待，发展有很多新要求，重任在肩，时不我待，让我们在市委的正确领导下，坚定信念不动摇，开拓进取不懈怠，埋头实干不松劲，为提前五年建成全面小康社会、建设现代化国际化城市而努力奋斗！
